# Glucocorticoids Impair the Airway Epithelial Barrier via Autophagy‐Associated Apoptosis in Asthma: A Preliminary Study Both In Vitro and In Vivo

**DOI:** 10.1002/pdi3.70060

**Published:** 2026-08-04

**Authors:** Hao You, Wenjing Zou, Tangqiaochu Gan, Yu Zhao, Jie Hu, Wen Tan, Ting Wang, Luo Ren, Zhengxiu Luo, Zhou Fu, Chao Niu

**Affiliations:** ^1^ Department of Respiratory Medicine, Children's Hospital of Chongqing Medical University National Clinical Research Center for Children and Adolescents' Health and Diseases Ministry of Education Key Laboratory of Child Development and Disorders Chongqing Municipal Health Commission Key Laboratory of Children's Vital Organ Development and Diseases Chongqing China; ^2^ Department of Pediatric Respiratory Medicine Chengdu Women's and Children's Central Hospital School of Medicine University of Electronic Science and Technology of China Chengdu Sichuan China; ^3^ Chongqing Key Laboratory of Child Rare Diseases in Infection and Immunity Chongqing China; ^4^ Key Laboratory of Children's Important Organ Development and Diseases of Chongqing Municipal Health Commission Chongqing China

**Keywords:** airway epithelium, apoptosis, asthma, autophagy, glucocorticoids

## Abstract

Glucocorticoid insensitivity affects a subset of asthma patients, yet its mechanism is unclear. Given the key role of airway epithelium in asthma, this study hypothesized that glucocorticoids may impair this barrier. We examined whether glucocorticoid‐induced autophagy‐associated apoptosis contributes to reduced treatment efficacy. Using an ovalbumin‐induced asthma model in BALB/c mice and 16HBE human bronchial epithelial cells treated with dexamethasone and/or autophagy inhibitor EACC (C_13_H_11_N_3_O_6_S_2_), we assessed cell viability and apoptosis via CCK‐8 and TUNEL assays, respectively. Apoptosis and autophagy markers were analyzed by quantitative polymerase chain reaction (PCR) and western blot. Dexamethasone alleviated airway inflammation but worsened epithelial integrity in asthmatic mice. It failed to downregulate pro‐apoptotic factors while reducing anti‐apoptotic factors both in vivo and in vitro. Dexamethasone decreased 16HBE cell viability and increased apoptosis. Mechanistically, dexamethasone upregulated autophagy markers and suppressed anti‐autophagy factors in mouse lungs and cells, concurrently reducing the phosphorylation of negative regulators of autophagy. Importantly, the autophagy inhibitor EACC enhanced cell viability and attenuated dexamethasone‐induced apoptotic signaling in 16HBE cells. These findings suggest glucocorticoids may compromise the airway epithelial barrier via autophagy‐associated apoptosis in vitro and in vivo, indicating autophagy inhibition as a potential hypothesis for future therapeutic exploration for glucocorticoid‐insensitive asthma.

## Graphical abstract

Mechanism underlying the negative effects of glucocorticoids on the airway epithelial barrier. Glucocorticoids upregulate autophagy factors and downregulate anti‐autophagy factors. Enhanced autophagy promotes excessive apoptosis and epithelial detachment, impairing airway barrier function. Barrier dysfunction perpetuates airway inflammation, establishing a vicious cycle of “autophagy/apoptosis—barrier damage—inflammation”.

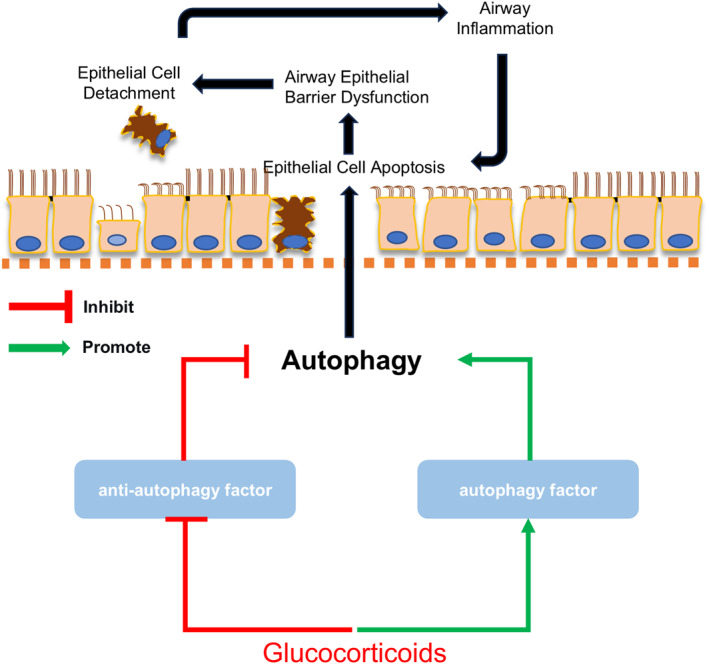

## 1 Introduction

Asthma, a chronic respiratory disorder characterized by persistent airway inflammation, hyperresponsiveness (AHR), and remodeling, clinically manifests as episodic dyspnea, wheezing, and coughing [1]. Current estimates indicate that asthma affects approximately 358 million people globally [2]. Moreover, consensus suggests that the prevalence will continue to rise, imposing a significant ongoing disease burden globally. Despite being the first‐line therapy for clinical asthma management with effective control in the majority, glucocorticoids fail to provide adequate control in a subset of patients: approximately 5%–10% exhibit a suboptimal response [3], the underlying mechanism of which remains unclear. This suboptimal response may be attributable, at least in part, to impairment of the airway epithelial barrier. The airway epithelium acts as the first defensive barrier of the respiratory system. Impairment of airway epithelium and reduced barrier function are nearly universal features of asthma and can be observed in almost all patients [4]. If airway epithelial integrity is impaired, inhaled toxins and pathogens gain easier access to the host, possibly causing an asthma attack. The integrity of the airway epithelial barrier in asthma is influenced by various factors. Despite the effective anti‐inflammatory effects, glucocorticoids are not purely beneficial to airway epithelial cells. Accumulating evidence indicates that glucocorticoids can injure the integrity of the airway epithelial barrier [5, 6, 7]. However, whether and how glucocorticoids directly compromise the epithelial barrier remains insufficiently explored. We hypothesize that glucocorticoids may injure this barrier by inducing epithelial cell apoptosis via the promotion of autophagy, thereby contributing to glucocorticoid resistance observed in some asthmatic patients.

Autophagy is a highly conserved catabolic process in eukaryotic cells. It targets abnormal protein aggregates, lipid droplets, and damaged organelles for lysosomal degradation, facilitating the recycling of metabolic components and playing a critical role in maintaining cellular homeostasis [8, 9]. Although moderate autophagy is considered a protective mechanism for maintaining cellular homeostasis, growing evidence points to an intricate crosstalk between autophagy and apoptosis, where the former can regulate and promote the latter under specific conditions [10, 11, 12, 13]. However, the direct impact of therapeutic doses (especially locally accumulated high concentrations) of glucocorticoids on autophagy in airway epithelium, as well as its consequential effect on epithelial cell fate and barrier function, remains poorly understood.

This study aims to investigate whether glucocorticoids reduce airway epithelial cell viability and impair airway epithelial barrier integrity by inducing autophagy‐associated apoptosis, thereby providing a theoretical foundation and new insights for improving the efficacy of asthma therapy.

## 2 Materials and Methods

### 2.1 Mice and Treatment

Six‐ to eight‐week‐old female BALB/c mice, purchased from the Experimental Animal Center of Chongqing Medical University, were housed under specific pathogen‐free conditions with controlled lighting (a 12‐h light/dark cycle) and provided a diet without ovalbumin (OVA). A total of 72 mice were used in these experiments, and these mice were randomly divided into four groups: normal control group (received phosphate‐buffered saline [PBS] only), asthma model group (OVA‐sensitized); low‐dose dexamethasone treatment group (1 mg/kg); high‐dose dexamethasone treatment group (5 mg/kg). The study was approved by the Ethics Committee of Chongqing Medical University (reference number: 2012023).

According to previously published methods with minor modifications [14, 15], mice were sensitized on days 0, 7, and 14 via intraperitoneal and subcutaneous administration of an emulsion comprising 100 μg OVA adsorbed to 2 mg Al(OH)_3_ in a total volume of 200 μL per mouse to establish an OVA‐induced allergic asthma model. Subsequently, during days 21–30, airway inflammation was elicited through daily 30‐min exposure to a 1% OVA aerosol. On the final three challenge days (days 28–30), mice assigned to treatment groups received an intraperitoneal injection of dexamethasone (1 or 5 mg/kg) 30 min prior to aerosol provocation. The asthmatic control group received an equivalent volume of PBS. At the end of the experiment, all mice were sacrificed by means of cervical dislocation.

### 2.2 Reagents and Antibodies

The following materials and reagents were used in this study: EACC (HY‐129111, MCE, Monmouth Junction, NJ, USA), dexamethasone (Meilunbio, MB1434‐2, Dalian, Liaoning, China), a CCK‐8 assay kit (Apexbio, K1018, Houston, TX, USA) and a TUNEL assay kit (Meilunbio, MA0223, Dalian, Liaoning, China).

Antibodies against p‐mTOR (CST, 2971, Danvers, MA, USA), mTOR (CST, 2972, Danvers, MA, USA), p‐PI3Kp85 (abcam, ab182651, Cambridge, Cambridgeshire, UK), PI3Kp85 (CST, 4257, Danvers, MA, USA), BAX (CST, 5023, Danvers, MA, USA), BCL‐2 (CST, 4223, Danvers, MA, USA) and GAPDH (CST, 2118, Danvers, MA, USA) were used for western blotting.

### 2.3 Noninvasive Measurement of Enhanced Pause (Penh)

The airway responsiveness to methacholine was quantified by the Penh index 24 hours post the final allergen challenge. For this assessment, unrestrained mice in a whole‐body plethysmograph (EMKA Technologies) were exposed to successively higher concentrations (0, 3.125, 6.25, 12.5, 25, and 50 mg/mL) of nebulized methacholine (acetyl‐β‐methylcholine chloride; Sigma‐Aldrich, A2251, St. Louis, MO, USA). The Penh value was subsequently recorded according to previously described methods and correlated with the degree of airflow obstruction [16, 17].

### 2.4 Bronchoalveolar Fluid Assays

Following sacrifice, the tracheas of mice were cannulated for bronchoalveolar lavage (BAL). The lungs were lavaged with six sequential 0.5 mL aliquots of PBS to collect BAL fluid.

After centrifugation of the fluid, a red blood lysis buffer (eBioscience 1 × RBC Lysis Buffer, ThermoFisher, 00‐4333‐57, Waltham, MA, USA) was added to remove the red blood cells. Total cell counts were determined using a hemocytometer under a microscope. For differential counts, at least 200 cells per sample were classified based on standard morphological and staining criteria [18]. The remaining BAL supernatant, obtained after a second centrifugation, was collected for subsequent cytokine analysis.

### 2.5 Cytokine Analysis

Commercially available enzyme‐linked immunosorbent assay kits for interleukin 5 (IL‐5; ≥ 8 pg/mL; 4A Biotech, Beijing, China) and interferon gamma (IFN‐γ; ≥ 8 pg/mL; Neobioscience, Shenzhen, Guangdong, China) were used to measure the concentrations of these cytokines in the BAL fluid.

### 2.6 Pathological Staining

Immediately following euthanasia, the tissues were harvested and fixed in 4% buffered formaldehyde, dehydrated in a graded alcohol series, cleared with dimethylbenzene, and embedded in paraffin. The paraffin blocks were serially sectioned at a thickness of 4 μm and stained with hematoxylin and eosin (H&E) using standard techniques.

### 2.7 Cell Culture and Grouping

Human bronchial epithelial cell line 16HBE cells were provided by the Chongqing Municipal Health Commission Key Laboratory of Children's Vital Organ Development and Diseases (Chongqing, China), and cultured in a cell incubator containing 5% CO_2_ at 95% humidity and 37°C. The cells were divided into the following groups: the control group, Dex groups and autophagy inhibitor groups. The concentrations of dexamethasone were selected based on relevant literature and the concentration of inhaled corticosteroids (ICSs) in the airway surface fluid layer, etc. (Supporting Information S1: Table S1).

To determine the appropriate concentrations, preliminary dose‐response experiments were performed. Based on the preliminary experiment, we set different concentrations of dexamethasone (5, 10 and 20 μmol/L) to interfere with 16HBE cells (Supporting Information S2: Figure S1A). We also chose 10 μmol/L dexamethasone plus 1 μmol/L EACC(C_13_H_11_N_3_O_6_S_2_), which seemed to be noncytotoxic, to interfere with 16HBE cells (Supporting Information S2: Figure S1B).

### 2.8 Cell Viability Assay

Following grouping and treatment as described in Section 2.7, 16HBE cells seeded in 96‐well plates were assessed for viability. At 24 hours after the drug intervention, a CCK‐8 kit was used. The measured absorbance directly correlates with the number of viable cells, serving as an indicator of cell viability.

### 2.9 TUNEL Assay

16HBE cells were seeded in 35 mm dishes and then grouped and treated as described in Section 2.8. Apoptosis was subsequently detected using a TUNEL assay kit according to the manufacturer's instructions. Apoptosis was subsequently examined under a laser scanning confocal microscope.

### 2.10 Real‐Time Quantitative Polymerase Chain Reaction (PCR)

Total RNA was isolated from16HBE cells, and cDNA synthesis was performed with a PrimeScript RT Reagent Kit (Takara, Otsu, Japan). Real‐time quantitative PCR was performed using standard techniques. The sequences of the primers are shown (Supporting Information S1: Table S2). For each sample, GAPDH were used as internal controls.

### 2.11 Western Blotting

Total cell lysates were obtained using a Total Protein Extraction Kit (KeyGen Biotech, KGB5303‐50, Nanjing, Jiangsu, China) according to the manufacturer's instructions. The proteins were separated by sodium dodecyl sulfate polyacrylamide gel electrophoresis (SDS‐PAGE) and then transferred onto polyvinylidene fluoride (PVDF) membranes. Afterwards, the proteins were probed with various primary antibodies and fluorophore‐labeled secondary antibodies. Finally, the blots were visualized using a fluorescence imaging system.

### 2.12 Statistical Analyses

Statistical analysis was performed using GraphPad Prism 6.0, and the data were presented as mean ± standard deviation. The two sets of data were analyzed using the *t* test, whereas multiple groups were analyzed using one‐way ANOVA. *p < 0*.*05* was considered to indicate statistical significance.

## 3 Results

### 3.1 A Mouse Model of OVA‐Induced Asthma Was Successfully Established

After the establishment of an OVA‐induced asthma model in BALB/c mice (Supporting Information S2: Figure S2A), a multi‐parametric approach confirmed its validity. Assessments including H&E staining (Supporting Information S2: Figure S2B), quantification of specific airway cytokines (Supporting Information S2: Figure S2C), counts of shed airway epithelial cells (Supporting Information S2: Figure S2C) and inflammatory cells in bronchoalvelar lavage fluid (BALF, Supporting Information S2: Figure S2D), and Penh measurements (Supporting Information S2: Figure S2E) all yielded results indicative of successful asthma induction.

### 3.2 Glucocorticoid Dexamethasone Alleviated Airway Inflammation but Failed to Alleviate Impairment to the Airway Epithelial Barrier in the Mice Asthma Model

An OVA‐induced asthmatic mouse model was generated and treated with dexamethasone to assess the effects of glucocorticoids on the airway epithelial barrier (Supporting Information S2: Figure S3).

**FIGURE 1:**
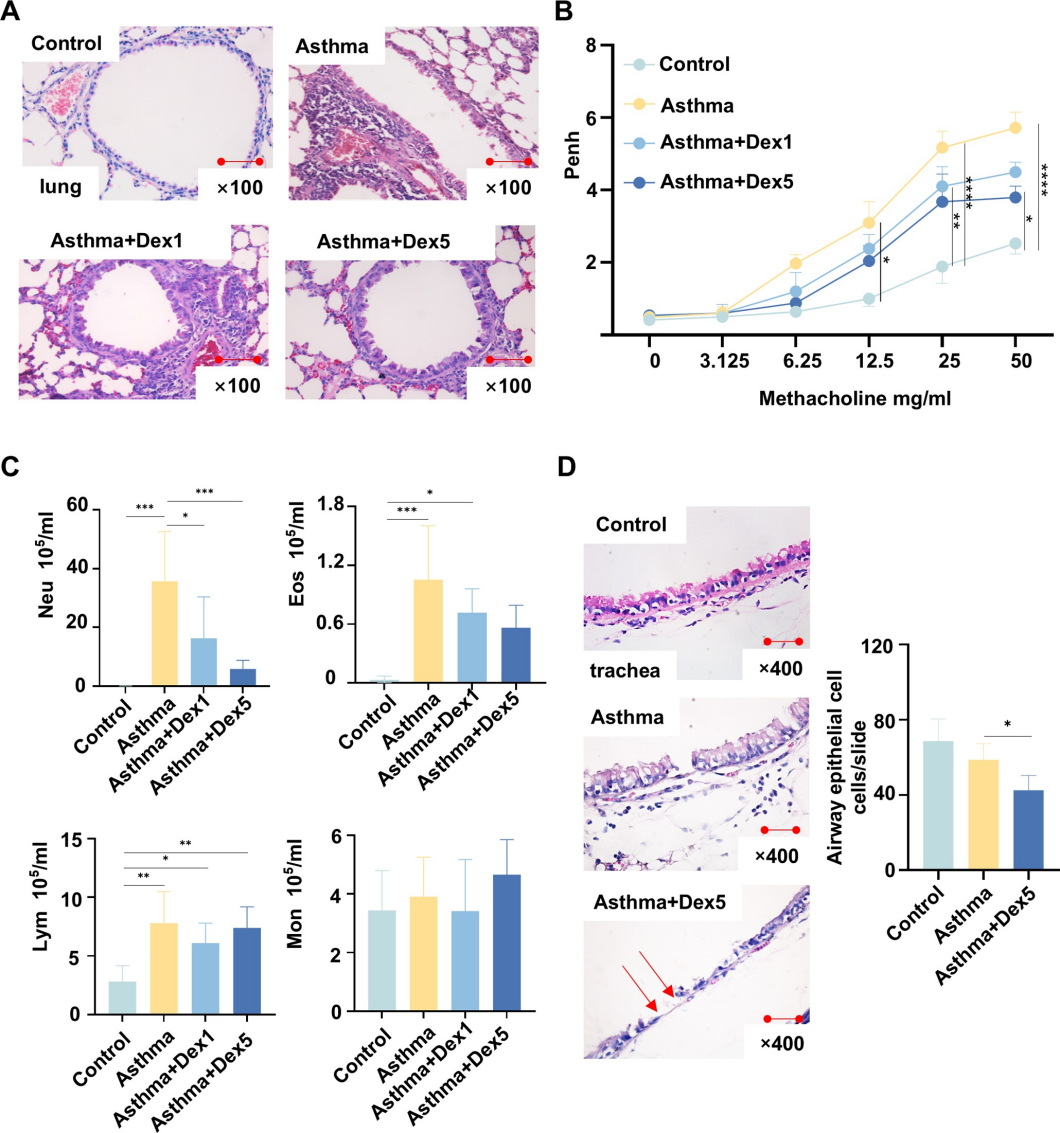
Dexamethasone alleviated airway inflammation but injured the airway epithelial barrier in a BALB/c mouse asthma model. (A) Representative hematoxylin and eosin (H&E)‐stained lung tissue sections. In the asthma model group, the number of inflammatory cells around the airways was markedly increased compared with the control. Dexamethasone treatment significantly alleviated this peribronchial inflammatory infiltration. (B) Airway hyperresponsiveness assessed by enhanced pause (Penh). Penh values were significantly elevated in asthma model mice, indicating increased airway hyperreactivity. Dexamethasone treatment substantially reduced Penh values toward normal levels. (C) Total inflammatory cell counts in bronchoalveolar lavage fluid (BALF). BALF from asthma model mice showed a significant increase in neutrophil, eosinophil and lymphocyte counts. Dexamethasone treatment effectively reduced the number of inflammatory cells in BALF. (D) Representative H&E‐stained trachea tissue sections and quantitative analysis of tracheal pathological specimens. In a BALB/c mouse asthma model, the airway epithelial barrier was injured, whereas dexamethasone seemed to enlarge the defect in the tracheal epithelium. The arrows indicate aggravated epithelial injury. *p < 0.05, **p < 0.01, ***p < 0.001 using one‐way ANOVA. Neu, neutrophil; Eos, eosinophil; Lym, lymphocyte; Mon, monocyte. Dex1, 1 mg/kg of dexamethasone; Dex5, 5 mg/kg of dexamethasone.

Compared with the asthma group, the asthma + Dex1 and asthma + Dex5 group showed significant improvements in asthma pathology evidenced by reduced airway inflammation on H&E staining (Figure 1A), decreased Penh values (Figure 1B), and lower inflammatory cell counts in BALF (Figure 1C). Interestingly, there was no statistically significant difference in macrophages among the four groups, which may be attributed to glucocorticoids primarily exerting functional inhibition on macrophages, rather than reducing their numbers [19]. However, a paradoxical finding was observed in the tracheal epithelium: despite significantly suppressing airway inflammation, dexamethasone exacerbated epithelial defects in OVA‐challenged mice, and the quantitative analysis of tracheal pathological specimens showed that the number of remaining epithelial cells was significantly lower in the asthma + Dex5 group (44.70 ± 8.59) than in the control group (67.29 ± 14.10) (Figure 1D).

### 3.3 Glucocorticoid Dexamethasone Modulated Apoptosis‐Related Gene Expression in Lung Tissue of Asthmatic Mice

To further verify the negative effect of dexamethasone on the airway epithelial barrier in the asthma mouse model, we used quantitative PCR to measure the levels of key factors involved in apoptosis in mouse lung tissue.

**FIGURE 2:**
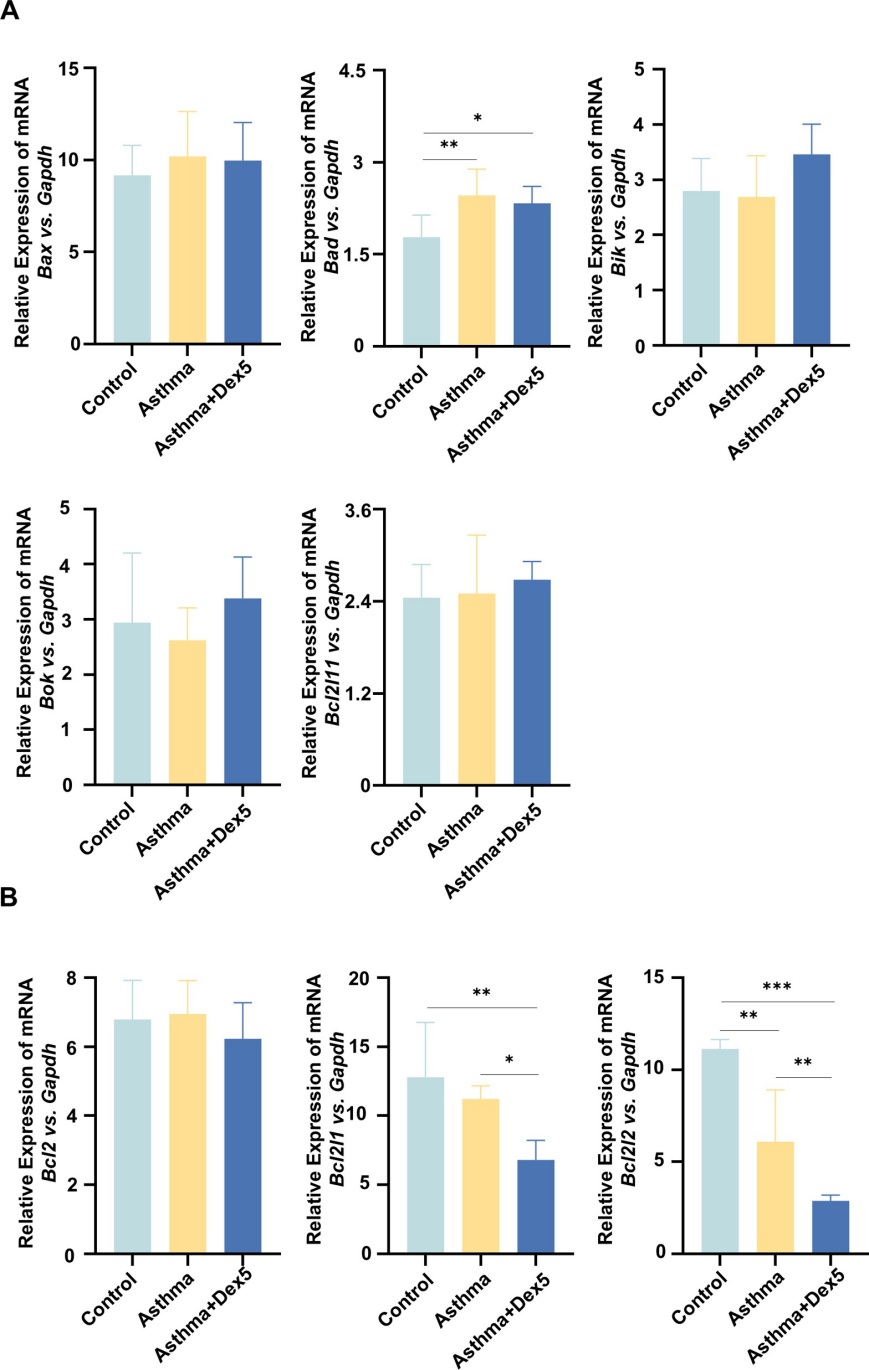
Dexamethasone modulated apoptosis‐related gene expression in lung tissue of asthmatic mice. (A) The mRNA levels of *Bax, Bad, Bik, Bok, and Bcl2l11*. Dexamethasone failed to downregulate mRNA levels of pro‐apoptotic factors. Specifically, although the mRNA expression levels of *Bad* in the Asthma + Dex5 group differs significantly from that in the control group, no statistically significant difference was observed when compared with the asthma group. (B) The mRNA levels of *Bcl2*, *Bcl2l1*, *and Bcl2l2*. Dexamethasone downregulated mRNA levels of *Bcl2l1 and Bcl2l2* compared to the control group, but failed to downregulate mRNA levels of *Bcl2* significantly. *p < 0.05, **p < 0.01, ***p < 0.001 (one‐way ANOVA). Dex5, 5 mg/kg of dexamethasone.

Compared with the asthma group, the mean mRNA levels of pro‐apoptotic factors—including *Bax, Bad, Bik, Bok, and Bcl2l11*—either failed to decrease to control levels or were elevated in the asthma + Dex5 group, with the most significant difference observed for *Bad* (2.33 ± 0.28 and 1.78 ± 0.36 in asthma + Dex5 group and control group respectively; *p* = 0.0149) (Figure 2A). In contrast, the mean mRNA levels of anti‐apoptotic factors, such as *Bcl2*, *Bcl* *2* *l* *1* and *Bcl* *2* *l* *2*, were lower in the asthma + Dex5 group than in the asthma group, with the difference being most significant for *Bcl2l2* (2.88 ± 0.31 in asthma + Dex5 group *vs.* 6.09 ± 2.82 in asthma group; *p* = 0.0039), followed by *Bcl2l1* (6.80 ± 1.43 in asthma + Dex5 group *vs.* 11.23 ± 0.94 in asthma group; *p* = 0.0404), while *Bcl2* did not show a significant difference (Figure 2B).

Due to the complex composition of lung tissue, these changes may reflect the combined response of multiple cells. To clarify the specific effect of dexamethasone on airway epithelial cells, we conducted subsequent in vitro experiments.

### 3.4 Glucocorticoid Dexamethasone Enhanced Autophagy‐Related Gene Expression in Lung Tissue of Asthmatic Mice

To further verify the negative effect of dexamethasone on airway epithelial barrier in an asthma mouse model, we used quantitative PCR to measure the levels of the factors involved in autophagy in mouse lung tissue.

**FIGURE 3:**
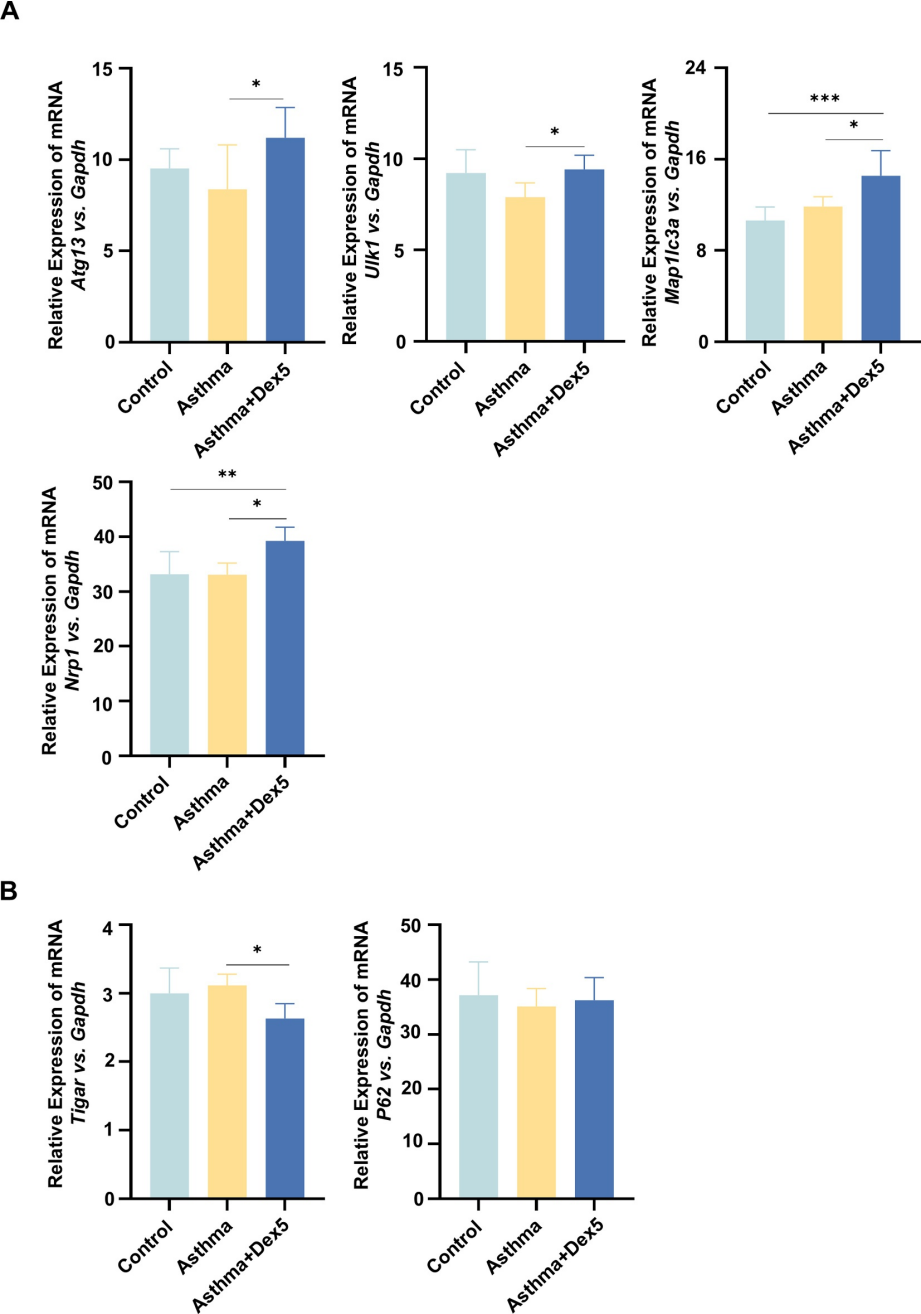
Dexamethasone may enhance autophagy‐related gene expression in lung tissue of asthmatic mice. (A) Dexamethasone may upregulate *Atg13, Ulk1, Map1lc3a*, and *Nrp1* mRNA levels in the Asthma + Dex5 group compared to the Asthma group. Notably, although *Map1lc3a* and *Nrp1* showed significant elevation relative to both Control and Asthma groups, *Atg13* and *Ulk1* levels did not differ significantly between Asthma and Control groups (*p* > 0.05), suggesting dexamethasone‐induced rather than asthma‐induced upregulation. (B) Dexamethasone downregulated the mRNA levels of *Tigar*, whereas that of *P62* was not affected. Dex5, 5 mg/kg of dexamethasone, *p < 0.05, **p < 0.01, ***p < 0.001 (one‐way ANOVA).

Compared with the asthma group, the mean mRNA levels of pro‐autophagy factors failed to recover to control levels or even increased in the asthma + Dex5 group, including *Atg13* (11.20 ± 1.66 in asthma + Dex5 group *vs.* 8.38 ± 2.42 in asthma group), *Ulk1* (9.42 ± 0.78 in asthma + Dex5 group *vs.* 7.89 ± 0.79 in asthma group), *Map1lc3a* [20] (14.56 ± 2.19 in asthma + Dex5 group *vs.* 11.85 ± 0.86 in asthma group) and *Nrp1* (39.25 ± 2.46 in asthma + Dex5 group *vs.* 33.07 ± 2.12 in asthma group) (Figure 3A). However, the mean mRNA levels of anti‐autophagy factor *Tig* *a* *r* [21] were decreased in the asthma + Dex5 group (2.63 ± 0.21 in asthma + Dex5 group *vs.* 3.12 ± 0.16 in asthma group) (Figure 3B).

To investigate autophagy specifically in epithelial cells, we next performed experiments in vitro.

### 3.5 Glucocorticoid Dexamethasone Decreased Cell Viability and Promoted Apoptosis in 16HBE Cells

A series of experiments in 16HBE cells were performed to assess the effects of dexamethasone on airway epithelial cell, including cells morphological analysis, CCK‐8 assays, TUNEL assays, and western blotting to evaluate cell viability and apoptosis.

**FIGURE 4:**
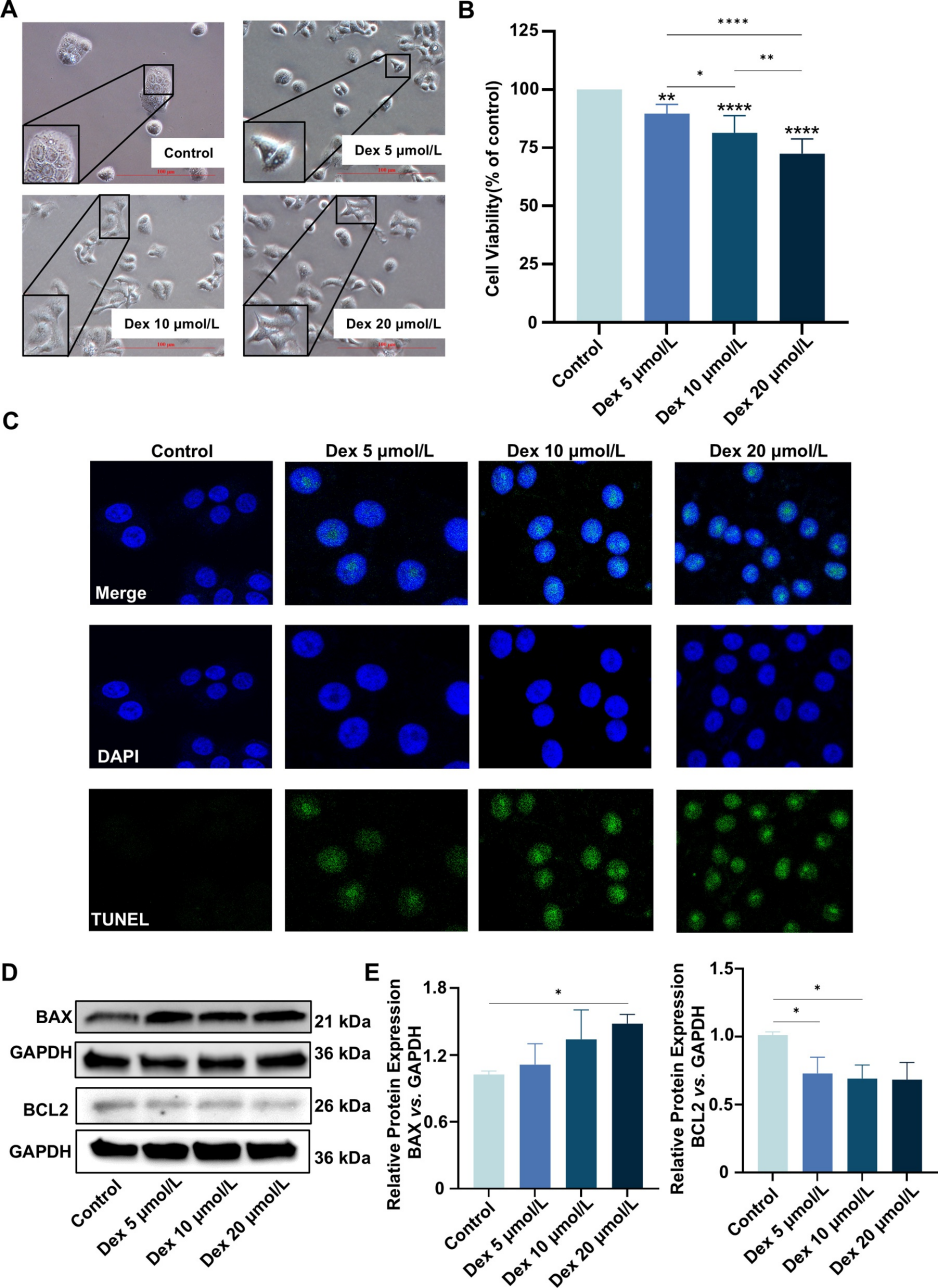
Dexamethasone decreased the viability and promoted the apoptosis of 16HBE cells. (A) Phase‐contrast images showing morphological changes in 16HBE cells after dexamethasone treatment. (B) Cell viability assessed by CCK‐8 assay. Dexamethasone treatment significantly reduced viability. (C) Apoptosis detected by TUNEL staining (green). Dexamethasone increased apoptotic cell numbers. (D) Representative western blots of BAX and BCL‐2. (E) Quantitative analysis of BAX and BCL‐2 protein expression. Dexamethasone upregulated BAX and downregulated BCL‐2. *p < 0.05, **p < 0.01, ***p < 0.001, ****p < 0.0001 (one‐way ANOVA).

Microscopic examination revealed that dexamethasone treatment induced dose‐dependent morphological abnormalities, including irregularity, pleomorphism, and increased bi‐/multinucleation, compared to the regular, well‐defined morphology of control cells. These observations suggest that glucocorticoids disrupt the normal morphology of airway epithelial cells (Figure 4A).

These morphological abnormalities were accompanied by dose‐dependent reduced viability measured by CCK‐8 assay (89.65% ± 3.93% in Dex 5 μmol/L *vs.* 81.37% ± 7.44% in Dex 10 μmol/L *vs.* 72.40% ± 6.40% in Dex 20 μmol/L) (Figure 4B) and increased apoptosis evaluated by TUNEL assays (Figure 4C). Given the role of BAX and BCL‐2 as key regulators of mitochondrial apoptosis [22], we analyzed their protein expression levels. Western blot analysis confirmed a shift toward apoptosis, with upregulated BAX in Dex 20 μmol/L group (*p* = 0.01) and downregulated BCL‐2 in Dex5 μmol/L group (*p* = 0.014) and Dex 10 μmol/L group (*p* = 0.020) (Figure 4D,E). These results indicate that the glucocorticoid dexamethasone decreased cell viability and promoted apoptosis in airway epithelial cells.

### 3.6 Glucocorticoid Dexamethasone Promoted Autophagy in Airway Epithelial Cells in 16HBE Cells

Autophagy consists of a series of steps, which can be broadly categorized into upstream signal integration primarily mediated by the mTORC1 complex, autophagosome formation, autophagosome‐lysosome fusion, and subsequent degradation of autophagic cargo [23, 24].

**FIGURE 5:**
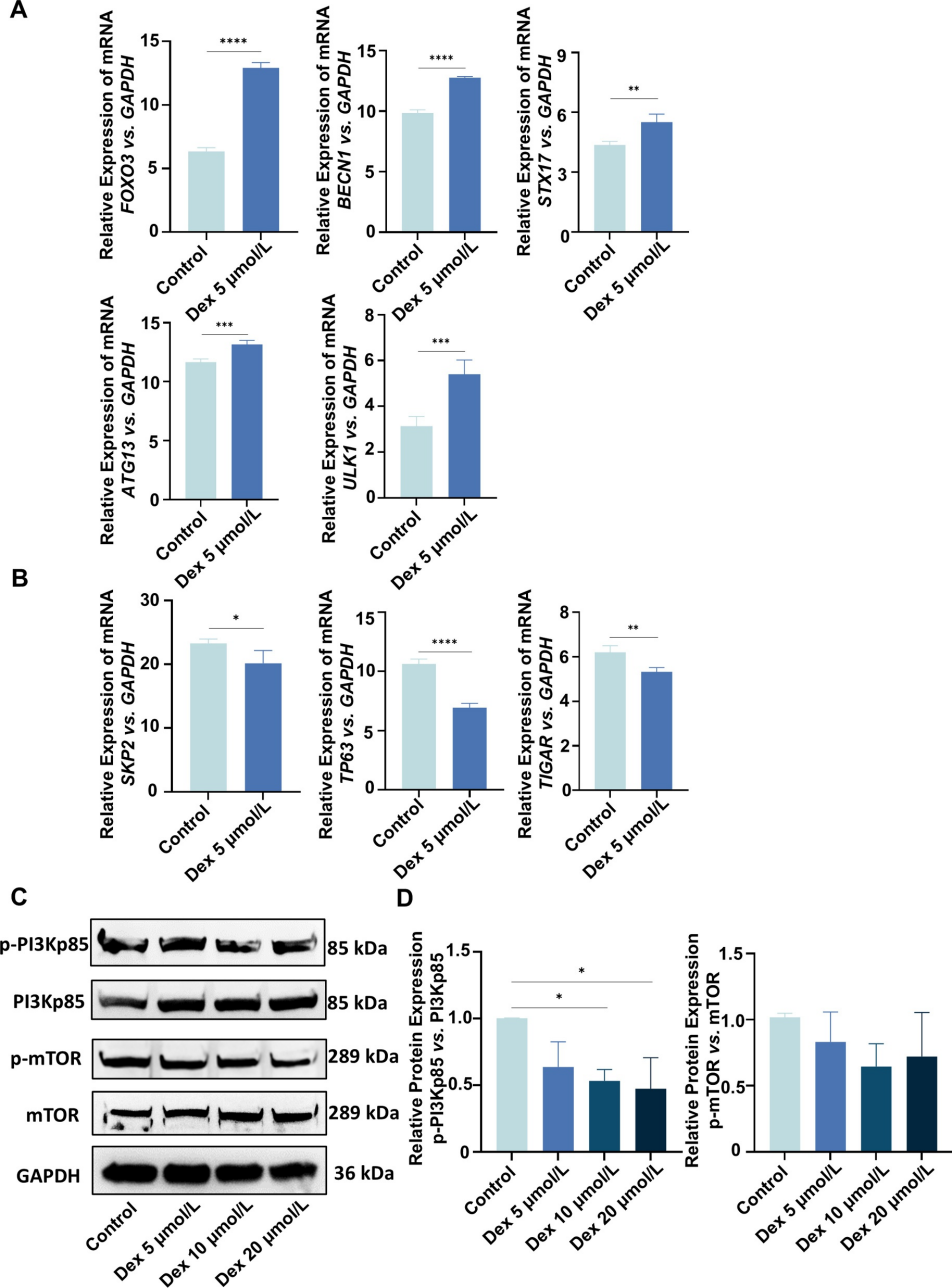
Dexamethasone promoted autophagy in 16HBE cells. (A) Dexamethasone upregulated mRNA levels of autophagy factors. (B) Dexamethasone downregulated mRNA levels of anti‐autophagy factors. (C) The protein expression levels of phosphorylation of PI3Kp85 and mTOR, key negative regulators of autophagy. *p < 0.05, **p < 0.01, ***p < 0.001, ****p < 0.0001 (*t* test for two groups; one‐way ANOVA for multiple groups).

To further verify the regulatory effect of dexamethasone on autophagy in airway epithelial cells, we used quantitative PCR to measure the levels of key factors involved in autophagy in 16HBE cells. The mRNA levels of positive regulators of autophagy were significantly upregulated in the Dex groups compared with the control groups (*FOXO3*: 12.93 ± 0.40 *vs.* 6.34 ± 0.30; *BECN1*: 12.77 ± 0.10 *vs.* 9.86 ± 0.26; *STX17*: 5.51 ± 0.40 *vs.* 4.37 ± 0.18; *ATG13*: 13.17 ± 0.33 *vs.* 11.66 ± 0.27; and *ULK1*: 5.41 ± 0.62 *vs.* 3.14 ± 0.42) (Figure 5A), whereas the opposite was observed for negative regulators (20.17 ± 2.00, 6.95 ± 0.36, 5.32 ± 0.19 for *SKP2*, *TP63* and *TIGAR* in Dex group respectively; 23.28 ± 0.67, 10.63 ± 0.42, 6.21 ± 0.29 for *SKP2*, *TP63* and *TIGAR* in control group respectively) (Figure 5B). Among these, the mRNA levels of *FOXO3*, *BECN1*, and *TP63* showed the most significant difference (*p* < 0.0001), followed by *ULK1* (*p* = 0.0009) and *ATG13* (*p* = 0.0004), and then *STX17* (*p* = 0.002), *TIGAR* (*p* = 0.002) and *SKP2* (*p* = 0.025).

Furthermore, Western blot analysis showed a significant reduction in the phosphorylation levels of PI3Kp85 across Dex 10 μmol/L group and Dex 20 μmol/L group compared to control groups (*p* = 0.026 and 0.014, respectively), whereas the phosphorylation levels of mTOR across all Dex‐dose groups showed no significant differences compared to control groups (Figure 5C and 5D). We believe these results indicate that the suppression of PI3K‐Akt‐mTOR signaling, which is known to inhibit autophagy, and the discrepancy in mTOR might be attributable to other non‐PI3K‐dependent mechanisms of mTOR activation.

In summary, the results suggest that the glucocorticoid dexamethasone increased autophagy in airway epithelial cells in vitro.

### 3.7 Glucocorticoid Dexamethasone Induced Apoptosis in 16HBE Cells by Promoting Autophagy, and Autophagy Inhibitor Attenuated Dexamethasone‐Induced Apoptosis

**FIGURE 6:**
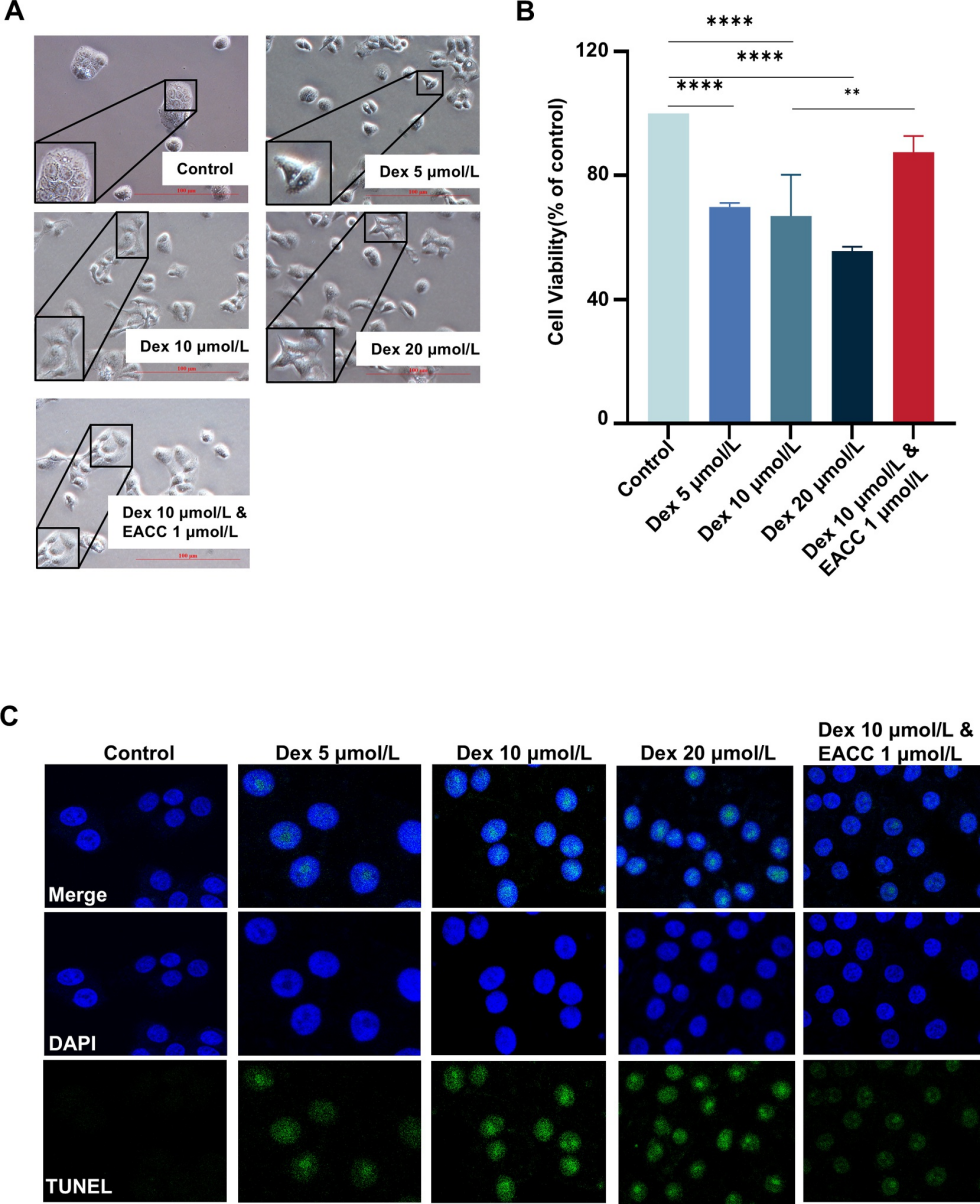
Autophagy inhibitor EACC reduced dexamethasone‐induced apoptosis of 16HBE cells. (A) Phase‐contrast images of cells treated with dexamethasone and EACC. (B) CCK‐8 assay showed that EACC partially restored dexamethasone‐reduced viability. (C) TUNEL assay showing that EACC reduced dexamethasone‐induced apoptosis. **p < 0.01, ****p < 0.0001 (*t* test for two groups; one‐way ANOVA for multiple groups).

We observed that dexamethasone treatment induced both morphological abnormalities and reduction of viability in 16HBE cells. These negative effects of dexamethasone could be attenuated by cotreatment with the autophagy inhibitor EACC evidenced by a partial reversal of morphological abnormalities (Figure 6A) and increased cell viability (66.92% ± 5.96% in the Dex 10 μmol/L group and 87.59% ± 2.30% in the Dex 10 μmol/L & EACC 1 μmol/L group) (*p* = 0.002) (Figure 6B). The TUNEL assay further indicated that Dex promoted apoptosis in a dose‐dependent manner, which was attenuated with the existence of the autophagy inhibitor EACC (Figure 6C).

Collectively, these findings suggest that autophagy inhibitors decrease glucocorticoid‐induced apoptosis in airway epithelial cells.

## 4 Discussion

Despite constituting the therapeutic cornerstone for asthma [25, 26], glucocorticoids offer neither a complete cure nor universal efficacy in asthma populations. Notably, a minority of asthma patients display relative insensitivity to glucocorticoids [26], and in a subset of these patients, inadequate response can escalate to glucocorticoid‐resistant asthma—a condition associated with heightened risks of acute exacerbation, recurrent hospitalization, and mortality [15, 27]. Our prior research has implicated glucocorticoids in potentially compromising the airway epithelial barrier [25]. This critical therapeutic limitation underscores an unmet clinical need and drives the need for a deeper investigation into the underlying mechanisms.

Our findings suggest that dexamethasone, a representative glucocorticoid, may impede airway epithelial barrier repair by reducing cell viability through mechanisms involving the induction of both autophagy and apoptosis. The observed reduction of cell viability is likely mediated by autophagy‐related apoptosis. The protective effect of EACC suggests that dexamethasone‐triggered apoptosis is autophagy‐associated. This crosstalk might involve autophagic degradation of anti‐apoptotic proteins or autophagosome‐associated activation of apoptotic pathways. These data may provide a plausible explanation for the suboptimal response to glucocorticoids in some asthma patients. Although the PI3K‐Akt‐mTOR signaling can also promote autophagy under certain conditions, such as nutrient starvation and myogenic differentiation [28, 29], all experiments in our study were conducted using epithelial cells under normal culture conditions, and so this possibility lies beyond the scope of the current investigation.

The negative effects of glucocorticoids on airway epithelial cells are primarily dose‐dependent at the tissue level. This dose‐dependency is dictated by the interplay between the delivered drug amount and the periciliary fluid volume. Human periciliary fluid forms a layer averaging 5–45 μm in depth [30, 31], with a total volume estimated between 1 and 10 mL throughout the whole airways [32, 33]. The pharmacokinetic profile of an inhaled 200 μg glucocorticoid dose, given a typical 10%–30% central airway deposition [32], yields a wide concentration range (2–100 μmol/L) in the airway. Additionally, the distribution of ICS is often uneven, causing localized epithelial regions to be exposed to excessively high concentration. This local over‐accumulation may underlie the unavoidable negative effects of ICS on the airway barrier. Moreover, the uneven distribution of inhaled glucocorticoids within the airways is not merely theoretical but has been evidenced by studies using various inhalation devices [34, 35].

In line with the prevailing view in the existing literature [14, 33], we agree that inhaled glucocorticoids can control asthma in most cases, in which their side effects on the airway epithelial barrier can be ignored. However, when glucocorticoids accumulate to excessive local concentration in the airway due to some conditions, their typically ignorable side effects may be amplified. These side effects could exacerbate injury to the airway epithelial barrier, which may compromise the therapeutic efficacy of glucocorticoids in asthma control.

The impairment of the airway epithelial barrier is a universal pathological feature of the disease [30]. Therefore, we propose a paradoxical effect of glucocorticoids to the airway epithelial barrier. Specifically, we hypothesize that despite the anti‐inflammatory effects of glucocorticoids, locally high concentrations of glucocorticoids caused by improper inhalation may decrease epithelial cell viability and excessively activate autophagy, disrupting normal cellular metabolism and impairing homeostasis. Concurrently, autophagy may decrease cell viability or even promote excessive apoptosis, further weakening the airway epithelial barrier. These negative effects of glucocorticoids, which are dose dependent, may ultimately diminish the ability of the host to defend against exogenous insults, thereby creating a vicious cycle in asthma management, potentially explaining why a subset of asthmatic patients exhibit a suboptimal response to long‐term inhaled glucocorticoid therapy.

This study has some limitations. Although this study suggests potential negative effects of glucocorticoids on the airway epithelium, its conclusions are constrained by the reliance on preclinical models and an absence of clinical evidence. The 16HBE cell line, merely a human bronchial cell line, differs from primary airway epithelial cells with respect to polarity and barrier function, and may not be able to fully represent the characteristics (such as polarity, tight junctions, mucus secretion, etc.) of all types of airway epithelial cells in vivo. This study did not cover other indicators of the airway epithelial barrier, such as trans‐epithelial resistance, permeability, and expression of tight junction proteins, which limits the persuasiveness of this research. This study did not verify whether the autophagy inhibitor could alleviate the airway epithelial barrier impairment caused by dexamethasone in the asthma animal model, which is a key aspect that needs to be confirmed in the future. The proposed link to autophagy also awaits direct evidence. Although this study found that autophagy inhibitors reduced apoptosis, which suggests an association between autophagy and apoptosis, it did not use autophagy inducers or knockdown of key autophagy genes to reverse and verify the causal chain of “autophagy/apoptosis—barrier impairment—inflammation.” Therefore, this study only provides preliminary evidence of association, and genetic tools (such as siRNA) could be used in future research to further verify this. Although systemic dexamethasone was utilized in this study to establish mechanistic proof‐of‐concept, we acknowledge that the pharmacokinetic profile differs substantially from inhaled corticosteroids. The local concentration range of 2–100 μmol/L estimated for inhaled glucocorticoids in airway surface liquid informed our in vitro dosing, but direct extrapolation to clinical inhalation therapy requires validation with clinically relevant formulations (e.g., budesonide, fluticasone) and delivery methods. Although our data suggest an autophagy‐associated mechanism, we acknowledge that definitive proof requires genetic validation (e.g., *ATG5*/*ATG7* knockdown) and multiple pharmacological inhibitors. A promising future direction involves exploring personalized asthma management strategies designed to reinforce airway epithelial barrier integrity.

Asthma is considered a major chronic respiratory disease worldwide, associated with substantial burden on the healthcare, increased individual costs, and impaired quality of life. Consequently, durable and effective disease control presents a persistent clinical and public health challenge. A novel mechanism is proposed herein, suggesting that glucocorticoid‐induced impairment of the airway epithelial barrier via autophagy‐associated apoptotic pathways may explain glucocorticoid resistance in asthma. To this end, we plan to utilize primary airway epithelial cells cultured at the air‐liquid interface or organoid models, together with in vivo animal models, to further validate and refine this hypothesis.

Our findings reveal the therapeutic paradox of glucocorticoids, providing a theoretical foundation for elucidating the mechanisms of glucocorticoid resistance and for developing improved clinical management strategies for asthma. In the future, it may be possible to identify individuals whose airway epithelia are susceptible to the toxicity of glucocorticoids by detecting the markers of airway epithelium or the state of shed epithelial cells in asthma patients. This would allow for early intervention or adjustment of treatment plans. Furthermore, exploring local delivery strategies, such as developing new inhalation devices that can ensure uniform drug distribution, or evaluating the potential synergistic effects and safety of combined inhalation of glucocorticoids and autophagy inhibitors in future studies, may provide new ideas for improving therapeutic efficacy.

## Author Contributions

**Hao You:** writing – original draft, validation, methodology, investigation, formal analysis. **Wenjing Zou:** methodology, investigation, formal analysis, data curation. **Tangqiaochu Gan:** investigation, formal analysis, data curation. **Yu Zhao:** validation, investigation. **Jie Hu:** validation, investigation, data curation. **Wen Tan:** methodology, validation. **Ting Wang:** methodology, investigation. **Luo Ren:** resources. **Zhengxiu Luo:** validation. **Zhou Fu:** supervision, funding acquisition. **Chao Niu:** writing – review and editing, supervision, project administration, funding acquisition, conceptualization.

## Ethics Statement

The study was approved by the Ethics Committee of Chongqing Medical University (reference number: 2012023).

## Conflicts of Interest

Chao Niu is a member of Pediatric Discovery Editorial Board. To minimize bias, he was excluded from all editorial decision‐making related to the acceptance of this article for publication. The other authors declare no conflicts of interest.

## Supporting information


Supporting Information S1

Supporting Information S2

## Data Availability

The data that support the findings of this study are available from the corresponding author upon reasonable request.
