# Supplementary material for: Glucocorticoids Impair the Airway Epithelial Barrier via Autophagy‐Associated Apoptosis in Asthma: A Preliminary Study Both In Vitro and In Vivo
Source: Pediatr Discov. 2026 Aug 4:e70060. Online ahead of print. doi: 10.1002/pdi3.70060 (PMC13439033; doi:10.1002/pdi3.70060)
Supplement: Supplementary file 1 [file PDI3-9999-0-s001.docx]

**Supplementary Table 1. The Estimated Concentration of Inhaled Corticosteroids in the Airway Surface Liquid**

| Common Asthma ICS^1^ Medications | ICS^1^ | Molecular Weight of ICS^1^ (g/mol) | Recommended inhalation of ICS (μg) | Volume of the ASL^2^ (ml) | Concentration^3^ (μmol/L) | 10-30% of concentration^4^ (μmol/L) |
| --- | --- | --- | --- | --- | --- | --- |
| Budesonide and Formoterol Fumarate Powder for Inhalation | Budesonide | 430.53 | 160 | 1-10 | 37.2- 371.659 | 3.7-111.5 |
| Fluticasone Propionate Inhaled Aerosol | Fluticasone | 500.6 | 100 | 1-10 | 20-200 | 2-60 |
| Salmeterol Xinafoate and Fluticasone Propionate Aerosol | Fluticasone | 500.6 | 100-500 | 1-10 | 20-100 | 2-30 |

^1^ ICS, inhaled corticosteroids.

^2^ ASL, airway surface liquid.

^3^ If an asthma patient inhales all the ICS into the airways, the concentration of ICS in the airway surface liquid would be...

^4^ If asthma patients inhale 10-30% of ICS into their airways, the concentration of ICS in the airway surface liquid would be...

Mass (g) = Concentration (mol/L) × Volume (L) × Molecular Weight (g/mol)

**Supplementary Table 2. The sequences (5’>3’) of the primers**

| Primer | forward | reverse |
| --- | --- | --- |
| FOXO3 | 5’-TACGTGGCCCCTGCAAAG -3’ | 5’-CTCCTCGGGGATCATGGAGT-3’ |
| BCL-1 | 5’-GGCGGAGGAGAACAAACAGA-3’ | 5’-GGAGGGCGGATTGGAAATGA-3’ |
| STX17 | 5’-CCGGCGGGAGGTTTTTCTAT-3’ | 5’-ATAGCTGGTTCAAGACGGCG-3’ |
| ATG13 | 5’-AGGCTTCCAGACAGTTCGTG-3’ | 5’-TGGGGTCCTCTCAAATTGCC-3’ |
| ULK1 | 5’-GTTCCAAACACCTCGGTCCT-3’ | 5’-TCCACCCAGAGACATCTTCCT-3’ |
| SKP2 | 5’-CTGAACATTTCAGTACTCTCGCA-3’ | 5’-GCAAAGTCTGCAGGGCAAAT-3’ |
| ΔNp63α | 5’-CCTGACCCTTACATCCAGCG-3’ | 5’-CTCTGGGACATGGTGGATCG-3’ |
| TIGAR | 5’-AGTTGTGAGTCACGGTGCTT-3’ | 5’-GCTCAGAGTGGCTGGTAAGG-3’ |
| Bax | 5’- GCTGGACACTGGACTTCCTC-3’ | 5’-GGGGTCCCGAAGTAGGAGAG-3’ |
| Bad | 5’-CTTAGAACTGGAGGGAGGAGGC-3’ | 5’-CTCACTCGGCTCAAACTCTGG-3’ |
| Bik | 5’-AGCTCAGCTTGGCAGAACAC-3’ | 5’-TCCATGAGGTCCACGTCTCT-3’ |
| Bok | 5’-GGTTCATGCCCTGGTTGACT-3’ | 5’-GAGGACATCCGTCCATCCAC-3’ |
| Bcl2l11 | 5’-GACCCGGCTTTGTTTCCCTT-3’ | 5’-AGAAGGTTGCTTGGCCATTTG-3’ |
| Bcl2 | 5’-AACATCGCCCTGTGGATGAC-3’ | 5’-AAACAGAGGTCGCATGCTGG-3’ |
| Bcl2l1 | 5’-AAAAGACCTTCCGGGGGTTG-3’ | 5’-GCTCACTTACTGGGTCTGCT-3’ |
| Bcl2l2 | 5’-CACTGGGTCCTAAGAGCTGC-3’ | 5’- CACTAGAGCCCGTGTGTCTG-3’ |
| Atg13 | 5’-TACAGAACTGCTGAGGACGC-3’ | 5’-TCACCACAGGAGTAGGGGTC-3’ |
| Ulk1 | 5’-GCCACCCTTTTCCTACCAGT-3’ | 5’-GCCCAGTGGTTCTTGGAGAG-3’ |
| Map1lc3a | 5’-TCGGGTTGCTCTTTTGGGTG-3’ | 5’-CAGACAGGCAAGGGCCTAAC-3’ |
| Nrp1 | 5’-ACTGGGCTGTGAAGTGGAAG-3’ | 5’-CACTGTGGCAGTTGGCCT-3’ |
| Tigar | 5’-ATCATTCAAGGACAAGGCGT-3’ | 5’-TGGAGAAGGCGTGGGTAAAC-3 |
| p62 | 5’-GATAGCCTTGGAGTCGGTGG-3’ | 5’-TCAGCCTCTGTAGATGGGTCC-3’ |
