# Supplementary material for: Glucocorticoids Impair the Airway Epithelial Barrier via Autophagy‐Associated Apoptosis in Asthma: A Preliminary Study Both In Vitro and In Vivo
Source: Pediatr Discov. 2026 Aug 4:e70060. Online ahead of print. doi: 10.1002/pdi3.70060 (PMC13439033; doi:10.1002/pdi3.70060)
Supplement: Supplementary file 2 [file PDI3-9999-0-s002.docx]

 **Figure S1 Toxicity of dexamethasone and EACC in 16HBE cells** (A) Toxicity of dexamethasone (Dex) to 16HBE cells, as determined by the CCK-8 assay. ***P* < 0.01, ****P* < 0.001, *****P* <0.0001 using one-way ANOVA. (B) Toxicity of the autophagy inhibitor EACC to 16HBE cells, as determined by the CCK-8 assay. ***P* < 0.01, *****P* <0.0001 using one-way ANOVA.

 **Figure S2 A mouse model of OVA-induced asthma was successfully established** (A) Establishment of an asthma model. (B) Representative hematoxylin and eosin (H&E)-stained lung tissue sections. (C) Analysis of the levels of representative airway cytokines and the number of shed airway epithelial cells in broncho-alveolar lavage fluid (BALF). (D) Airway hyperresponsiveness, as assessed by enhanced pause (Penh) values. **P* < 0.05, ***P* < 0.01 using t tests. (E) Total inflammatory cell counts in the BALF.

 **Figure S3 Establishment and grouping of the OVA-induced asthma mouse models** The mice were sensitized with both 50 µg of OVA and 1 mg of aluminum hydroxide (alum) via intraperitoneal (i.p.) injection and 50 µg of OVA and 1 mg of alum via hypodermic injection (i.h.) at the roots of the extremities on days 0, 7 and 14. The mice were exposed to 1% aerosol of OVA for 30 min beginning on day 21 for 10 days. Beginning on day 28, the mice received intraperitoneal injections of Dex at different concentrations for three days. After day 30, the mice were sacrificed, and lung and tracheal tissues were collected for analysis.
